# A Spatiotemporal Model of CXCL10 as a Master Regulator of Immune Evasion and Metastasis in Osteosarcoma

**DOI:** 10.3390/ijms27010319

**Published:** 2025-12-27

**Authors:** Benjamin B. Gyau, Tsz-Kwong Man

**Affiliations:** 1 Section of Hematology and Oncology, Department of Pediatrics, Baylor College of Medicine, Houston, TX 77030, USA; 2 Texas Children’s Cancer and Hematology Center, Houston, TX 77030, USA; 3 Dan L. Duncan Comprehensive Cancer Center, Baylor College of Medicine, Houston, TX 77030, USA

**Keywords:** osteosarcoma, tumor microenvironment, chemokine signaling, CXCL10, CXCR3, immune cell trafficking, pre-metastatic niche, inflammation, immune evasion, metastasis

## Abstract

The C-X-C motif chemokine ligand 10 (CXCL10) is implicated in the progression of osteosarcoma (OS), the most aggressive pediatric bone malignancy. However, its role often presents a profound clinical paradox: although high circulating levels are strongly linked to poor prognosis, its canonical function is to recruit anti-tumor immune cells. This review unravels these contrasting roles by proposing a novel spatiotemporal model. We argue that in the early stages, immune-evading OS cells initiate the formation of a pre-metastatic niche (PMN) in the lungs, creating a localized inflammatory environment that becomes the primary source of elevated circulating CXCL10. As the disease progresses, elevated systemic levels of CXCL10 overwhelm the localized chemokine gradient at the primary tumor site, creating a potent immune decoy that diverts anti-tumor CXCR3+ T cells away from the tumor. The resulting immune desertification permits unchecked tumor growth and an increased metastatic burden. We also discuss the therapeutic implications of this model, proposing that disrupting the chemokine axis offers a roadmap for developing rational, stage-specific therapies to effectively combat metastatic OS.

## 1. Introduction

Metastasis remains the leading cause of mortality for most cancers, transforming a localized and manageable disease into a systemic, often fatal challenge [1,2,3]. This is especially true for osteosarcoma (OS), the most common primary bone malignancy in adolescents, where the 5-year survival rate drops from around 70% for localized disease to a stark 20–30% for patients with metastatic disease [4,5,6]. Significant progress has been made in unraveling the complex, multi-step metastatic cascade, which involves genomic instability endowing tumor cells (the “seed”) with migratory capacity, the critical formation of the pre-metastatic niche (PMN) where the primary tumor primes distant organs (the “soil”) for colonization, along with local invasion, intravasation, circulation survival, and distant organ extravasation and adaptation [7,8,9]. In OS, successful navigation of this cascade is intricately governed by a dynamic and reciprocal interaction with the tumor microenvironment (TME)—a heterogeneous ecosystem dominated by myeloid cells and characterized by an immune-cold, anti-tumor suppressive milieu structured by a complex network of signaling molecules [10,11,12,13,14,15,16,17].

Central to modulating cellular activities and trafficking within the TME are chemokines, a family of small cytokines that significantly influence cellular functions such as proliferation, adhesion, and migration [18,19]. The C-X-C motif chemokine ligand 10 (CXCL10) has emerged as a key regulator with profoundly dual and contrasting roles. On one hand, strong clinical evidence from multiple independent cohorts demonstrates that high circulating CXCL10 levels in the serum of OS patients at diagnosis serve as a significant and independent predictor of metastatic disease and poor overall survival, suggesting a pro-tumorigenic, pro-metastatic function [19,20,21,22]. On the other hand, the canonical role of CXCL10 is to act as a potent chemoattractant for anti-tumor immune cells, including activated T cells and natural killer (NK) cells, via its receptor CXCR3 within the TME [23,24,25,26,27,28]. Indeed, analysis of OS tumor databases reveals that high intra-tumoral expression of CXCL10 and/or CXCR3 correlates with improved prognosis, consistent with a robust local anti-tumor immune response [22,29,30,31,32]. This apparent contradiction reframes the central question from a simplistic good-versus-bad dichotomy into a complex spatiotemporal puzzle: how does high systemic CXCL10—an indicator of poor prognosis—arise, and by what mechanism does it subvert the established anti-tumor functions of the local CXCL10-CXCR3 axis?

This review provides an overview of the diverse roles of CXCL10 in the metastatic cascade, highlighting its direct pro-migratory effects on OS cells, its critical involvement in conditioning the lung PMN, and its role in facilitating the adaptation of circulating OS cells to new environments. We discuss how these early, localized events lead to elevated systemic CXCL10 levels, which shift from being mere a biomarker of ongoing metastasis to an active mediator of immune evasion. We propose that high systemic tumor-derived CXCL10 establishes an immune decoy, misdirecting potent anti-tumor T cells away from the primary tumor. This mechanism drives immune desertification or immune-cold state at the primary site, enabling uncontrolled tumor growth and enhanced metastatic seeding, ultimately manifesting in overt metastases associated with late-stage disease and poor prognosis. Given the stagnant progress in improving survival rates for metastatic OS over the past three decades, there is an urgent need for therapies that effectively target and suppress metastasis. This review discusses the significant therapeutic implications of our proposed framework, suggesting that a better understanding of the CXCL10 axis could pave the way for novel interventions aimed at blocking its pro-metastatic actions, disrupting the systemic CXCL10 gradient, and harnessing its immunogenic potential to combat metastatic OS.

## 2. The CXCL10 Signaling Network

### 2.1. The CXCL10-CXCR3 Axis: The Canonical Signaling Pathway in Cancers, OS and Immune Modulation

Members of the C-X-C motif chemokine family play central roles in tumor development and immune modulation across various cancers [33,34,35,36,37,38]. Structurally, the chemokine family is divided into two groups based on the presence or absence of a glutamic acid-leucine-arginine (ELR) motif. ELR-positive chemokines, including CXCL1, CXCL5, and CXCL8, are generally pro-tumorigenic by promoting angiogenesis and recruiting pro-tumor immune cells [39,40,41,42]. For example, in pancreatic adenocarcinoma, NF-κB-driven expression of CXCL5 fosters tumor progression via recruitment of CXCR2^+^ myeloid-derived suppressor cells (MDSC) and tumor-associated neutrophils [43,44]. Similarly, the CXCL12-CXCR4 axis drives invasion and distant-organ metastasis in breast and pancreatic cancers [45,46,47]. In OS, the CXCL1-CXCR2 axis is critical for lung metastasis by enhancing OS cell motility and increasing vascular cell adhesion molecule 1 (VCAM-1) expression [48,49].

**Figure 1:**
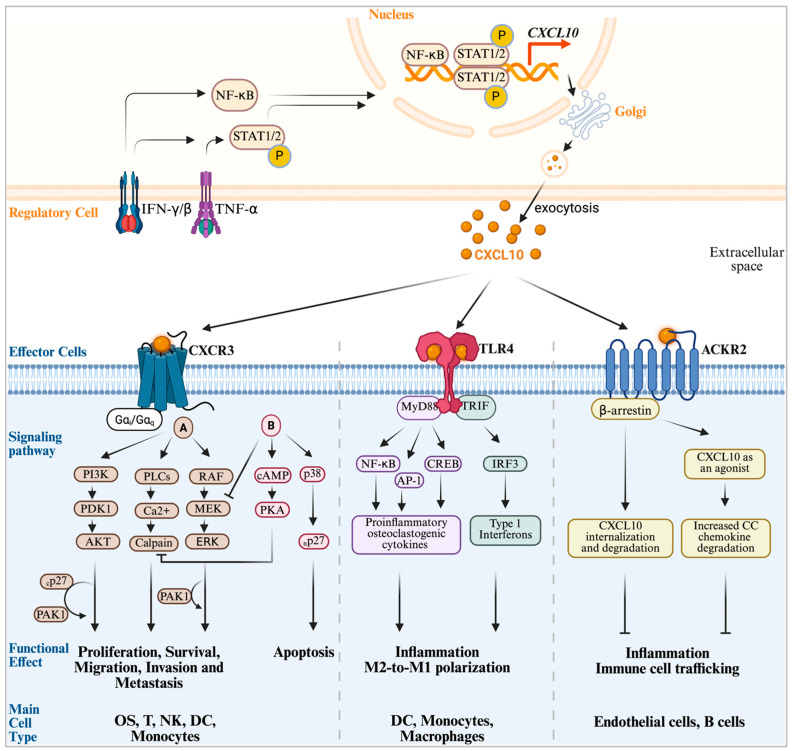
Canonical and non-canonical signaling networks of CXCL10. CXCL10 production in regulatory cells—including monocytes, fibroblasts, and OS cells—within the TME is primarily induced by pro-inflammatory cytokines such as IFN-γ, IFN-β, and TNF-α. These stimuli activate intracellular signaling cascades that culminate in the phosphorylation and nuclear translocation of the transcription factors NF-κB and STAT1/2, driving CXCL10 gene transcription. The translated CXCL10 protein is processed through the Golgi apparatus and secreted into the extracellular milieu via exocytosis. Secreted CXCL10 interacts with multiple receptors on adjacent effector cells, initiating diverse signaling pathways and downstream functional responses. CXCL10–CXCR3 canonical signaling: CXCL10 binds to its cognate G protein–coupled receptor CXCR3, which is expressed on OS cells, T cells, NK cells, and monocytes. Ligand engagement of CXCR3-A activates pro-survival and metastatic pathways, notably the PI3K–PDK1–AKT axis. In contrast, CXCR3-B engagement promotes apoptosis and suppresses survival signaling through the p38 MAPK and cAMP-dependent pathways. CXCL10 non-canonical signaling: CXCL10 also signals through non-canonical receptors, particularly TLR4 and ACKR2, which elicit opposing effects on inflammation. CXCL10–TLR4 interaction engages MyD88-dependent pathways leading to NF-κB-, AP-1-, and CREB-mediated transcription of pro-inflammatory cytokines, while IRF3 activation induces type I interferon production. Conversely, ACKR2 functions as a scavenger receptor: β-arrestin–mediated binding of CXCL10 to ACKR2 promotes its internalization and lysosomal degradation. This clearance mechanism is essential for shaping chemokine gradients, thereby attenuating inflammation and restricting immune cell trafficking within the TME. Standard arrow conventions apply. Created in Biorender. Benjamin B Gyau. (2025) https://app.biorender.com/illustrations/68c44a55e0acb9484c289e06.

In contrast, ELR-negative chemokines such as CXCL9, CXCL10, and CXCL11 are classically viewed as anti-tumor agents due to their angiostatic and immune modulatory effects mediated by their shared receptor, CXCR3 [39,50]. CXCR3, a high-affinity G protein-coupled receptor, mediates paracrine signaling primarily via CXCL10 and plays a vital role in regulating the migration, differentiation, and activation of immune cells—including cytotoxic T lymphocytes (CTL), NK cells, and monocytes—within the OS TME (Figure 1, Lower panel, left) [26,51,52,53]. Under interferon-γ (IFN-γ) influence, CXCL10-CXCR3 signaling also recruits regulatory T cells, suggesting context-dependent regulation of this axis [54]. Despite its well-characterized roles in inflammation and immune regulation, the CXCL10-CXCR3 axis is paradoxically implicated in malignant growth and progression in several tumors, including OS, underscoring its dual functionality [53,55]. This duality is central to the paradox in OS wherein high circulating CXCL10 levels correlate with poor prognosis, yet elevated intra-tumoral expression aligns with improved outcomes.

Current evidence suggests that CXCL10’s functional effects depend on spatiotemporal context—including local versus systemic concentration, cellular source (tumor versus stromal), and the specific CXCR3 receptor isoforms expressed. CXCL10 signaling is intricately regulated by three CXCR3 splice variants: CXCR3-A, CXCR3-B, and CXCR3-alt. While CXCR3-alt’s function remains poorly understood, CXCR3-A and CXCR3-B exhibit opposing effects that often govern cellular fate in cancer [56]. CXCR3-A is generally pro-tumorigenic in the context of cancer cell expression [57,58,59], and it is the canonical isoform expressed on activated T lymphocytes and NK cells [60,61,62]. Upon CXCL10 binding, it triggers Gαi-protein coupled signaling that drives calcium mobilization and the PI3K/AKT and MAPK/ERK signaling cascades necessary for the polymerization of actin and the cytoskeletal reorganization required for cellular migration/chemotaxis [63,64,65]. Conversely, CXCR3-B mediates anti-proliferative and angiostatic signals—a conserved paradigm observed across multiple cancer types [66,67,68]. Table 1 summarizes the distinct signaling, cellular distribution, and functional roles of these isoforms, highlighting their opposing contributions to OS progression.

Studies demonstrate that malignant breast cancer cells, particularly aggressive basal types, express a higher CXCR3-A to CXCR3-B ratio compared to predominantly CXCR3-B-expressing non-malignant mammary epithelial cells [69]. A similar pattern is seen in renal cell carcinoma and correlates with tumor grade [70]. A similar paradigm occurs in OS, where the isoform balance critically influences metastatic potential. Rescue experiments in CXCR3 knockout OS cells show that re-expression of CXCR3-A, but not CXCR3-B, fully restores migratory and metastatic phenotypes, establishing CXCR3-A as the isoform driving CXCL10’s pro-metastatic signaling [71]. Similarly, infiltrating immune effector cells within these lesions predominantly express the chemotactic CXCR3-A isoform, facilitating their recruitment, although their function may be compromised by the immunosuppressive TME [60,61].

### 2.2. The CXCL10-TLR4 Axis, a Non-Canonical Innate Immune Inflammatory Signaling Pathway

Toll-Like Receptor 4 (TLR4) is a pivotal pattern recognition receptor in the innate immune system. Emerging evidence reveals that CXCL10 can act as a non-canonical ligand for TLR4, establishing a distinct signaling paradigm separate from the well-known chemotactic functions mediated by CXCR3 (Figure 1, Lower panel, middle) [72,73,74]. A landmark study in rheumatoid arthritis models elegantly delineated the differential roles of these two receptors, demonstrating that CXCL10-induced migration of macrophages and T cells is exclusively CXCR3-dependent, whereas induction of pro-inflammatory and osteoclastogenic cytokines such as RANKL, TNF-α, and IL-6 requires cooperative engagement of both CXCR3 and TLR4 [75]. This cooperative signaling plays a crucial role in macrophage polarization. In cancers like pancreatic ductal adenocarcinoma, CXCL10-CXCR3 signaling maintains an inflammatory, anti-tumor M1 macrophage phenotype, and blockade of this axis promotes a shift to a tumor-supportive M2 phenotype [25]. In OS, where the TME is often enriched with immunosuppressive M2-like tumor-associated macrophages (TAM) [17,76], TLR4 activation emerges as a potent stimulus to reprogram these cells toward a pro-inflammatory, anti-tumor M1 phenotype. This was recently demonstrated in preclinical OS models where systemic administration of a detoxified TLR4 agonist inhibited tumor growth and lung metastasis, effects that depended on recruitment and activation of CD8^+^ T cells [77]. Thus, the CXCL10-TLR4 axis constitutes an endogenous mechanism for activating potent innate anti-tumor immunity within the OS TME.

### 2.3. The CXCL0-ACKR2 Axis: An Atypical Scavenger and Chemokine Gradient Modulator

Maintaining chemokine concentration gradients and regulating the magnitude and duration of inflammatory responses are essential for tissue homeostasis. ACKR2, also known as D6, is a member of the atypical chemokine receptor (ACKR) family that primarily functions as a scavenger receptor [78]. It binds ligands with high affinity, internalizes them, and directs their degradation, thereby modulating chemokine availability (Figure 1, Lower panel, right). While ACKR2 is traditionally recognized for binding most inflammatory CC-family chemokines, recent evidence has identified CXCL10, an inflammatory CXC chemokine, as a robust agonist and ligand of ACKR2 [79]. The role of ACKR2 in cancer is complex and highly context-dependent. For instance, in inflammation-driven tumor models, the absence of ACKR2 leads to increased tumor growth linked to unchecked inflammation [80]. Conversely, in melanoma and breast cancer models, ACKR2-deficient mice exhibit protection against lung metastasis [81]. This apparent paradox was mechanistically addressed by a study demonstrating that ACKR2 is uniquely expressed by blood endothelial cells of lung capillary aerocytes. Selective deletion of ACKR2 in these cells enhances T lymphocyte extravasation into the lungs, reducing lung metastases [82]. This finding positions ACKR2 as a critical gatekeeper of immune cell trafficking in the lungs with significant therapeutic implications.

In the context of OS, targeting the CXCL10-ACKR2 axis may offer a novel strategy for lung-directed therapies aimed at modulating immune cell migration and metastatic seeding. ACKR2’s constitutive scavenging activity also serves as a fine-tuner of chemokine gradients, thereby shaping inflammatory environments and immune cell localization [83,84,85]. Its regulatory function involves continuous ligand internalization and recycling, enabling rapid responses to chemokine fluctuations without requiring transcriptional changes. The modulation of CXCL10 availability by ACKR2 thus represents an important layer of control in the immune landscape of the TME and metastatic niches. It is biologically plausible that a systemic or local downregulation of ACKR2 activity—whether through genetic mutation, epigenetic silencing, or saturation by excess ligand—contributes to the pathologically high serum CXCL10 levels observed in metastatic OS patients [86,87]. A failure of this scavenging mechanism would exacerbate the systemic immune decoy effect by allowing chemokine levels to remain elevated in the circulation, preventing the establishment of precise chemotactic gradients required for T-cell homing.

### 2.4. The CXCL10-CXCR3 Downstream Signaling Pathways in Cellular Activity Modulation

CXCL10 signaling in immune and cancer cells activates multiple intracellular pathways that regulate cell survival, proliferation, and motility. In immune cells, the PI3K/AKT and JAK/STAT1 pathways play pivotal roles in CXCL10-CXCR3-mediated trafficking, activation, and the production of cytolytic molecules like perforin and granzyme B [88,89,90]. In colon cancer cells, activation of the CXCL10-CXCR3 axis stimulates the PI3K/AKT pathway, suppressing GSK-3β phosphorylation and upregulating the transcription factor Snail, a key regulator of epithelial–mesenchymal transition (EMT), thereby promoting proliferation, survival, invasion, and metastasis [91]. Similar downstream signaling activation has been documented in prostate [92], breast [93], melanoma [94], and gastric [95] cancers.

In OS, canonical pathways including PI3K/AKT and MAPK/ERK mediate the pro-tumorigenic effects of CXCL10-CXCR3 signaling [71,96,97,98]. Our lab has shown that CXCL10 induces rapid phosphorylation of AKT at serine 473 in OS cells, an effect that is completely abolished in CXCR3-knockout cells, providing a direct mechanistic link. Furthermore, CXCL10-induced activation of the AKT pathway in OS is closely connected to the p21-activated kinase 1 (PAK1) pathway, a critical regulator of metastatic potential [71]. PAK1 governs cell motility, proliferation, and survival, with its dysregulation linked to aggressive tumor phenotypes and metastasis in various cancers including breast, lung, and hepatocellular carcinoma [99,100,101]. Our previous studies showed mislocalization of p27 to the cytoplasm in OS, where it interacts with and activates PAK1, enhancing pulmonary metastasis. Silencing PAK1 in OS cells with mislocalized p27 abrogated metastatic behavior in vivo [102]. Interestingly, a kinase-dead PAK1 mutant paradoxically increased pulmonary metastasis, likely through compensatory pathways or kinase-independent scaffolding functions mediated by increased phosphorylation at threonine 423 (T423) [103]. Given AKT’s role in modulating p27 cytoplasmic localization [104,105,106,107], these findings suggest a complex interplay among AKT, PAK1, and cytoplasmic p27 in OS progression.

## 3. CXCL10’s Pro-Metastatic Arm: The Role of CXCL10 in OS Initiation, Growth and Early-Stage Metastasis

### 3.1. CXCL10 in OS Initiation

Although direct evidence linking CXCL10 to the initiation of OS remains limited, accumulating data implicate the CXCL10–CXCR3 axis as a critical regulator of the bone niche, particularly through its involvement in bone remodeling—a dynamic cycle of bone resorption by osteoclasts and formation by osteoblasts. Osteoblasts can be stimulated to produce CXCL10 in response to inflammatory signals such as bacterial lipopolysaccharide (LPS) or proinflammatory cytokines, including TNF-α, positioning them as sensors of local inflammation capable of orchestrating chemokine-mediated responses [108,109].

Beyond osteoblasts, the CXCL10–CXCR3 axis also contributes to osteoclastogenesis, the differentiation and activation of osteoclasts that drive bone resorption. In a pivotal study, Lee et al. demonstrated that CXCL10 not only recruits CXCR3-expressing cancer cells to bone metastases but also promotes osteoclast differentiation [110]. RANKL—the master cytokine regulating osteoclast formation—induces osteoclast precursors to secrete CXCL10 [111]. Under persistent inflammatory conditions, such as chronic TNF-α exposure, CXCL10 amplifies T-cell recruitment to the bone microenvironment, further enhancing RANKL expression and signaling. This feedback loop drives excessive osteoclast activation and disrupts normal bone homeostasis. Notably, CXCL10 neutralization significantly reduced osteoclast numbers and bone resorption in vivo, underscoring its central role in maintaining this pathological cycle [110]. Chronic CXCL10-driven bone turnover may thus generate a pro-tumorigenic microenvironment conducive to OS initiation.

Persistent CXCL10-mediated bone resorption also releases growth factors stored within the bone matrix, exposing resident mesenchymal stem cells (MSC) to mitogenic stimuli and proliferative stress during repair [112,113]. This sustained stimulation could heighten the risk of malignant transformation. Given that OS originates from dysregulated osteogenesis—where aberrant differentiation of pluripotent MSC into osteoblasts is driven by oncogenic disruptions in pathways such as PI3K, Wnt/β-catenin, and Notch—the CXCL10–CXCR3 axis may act as a non–cell-autonomous driver of tumorigenesis within an inflamed bone microenvironment [114,115,116].

Although the direct influence of CXCL10 on MSC osteogenic differentiation remains unclear, insights from hematopoietic stem cell (HSC) biology provide compelling parallels. The CXCL10–CXCR3 axis maintains HSC quiescence and regulates lineage commitment; genetic ablation of CXCL10 induces HSC cycling and skews differentiation toward the B-cell lineage at the expense of myeloid cells [117]. A similar mechanism may operate in MSC, whereby chronic CXCL10–CXCR3 signaling perturbs the delicate balance between proliferation and differentiation. This may favor proliferative expansion over osteogenic maturation, leading to the differentiation arrest characteristic of OS. In MSC harboring oncogenic mutations such as TP53 and RB1 loss, persistent inflammatory pressure via the CXCL10–CXCR3 axis could provide the necessary “second hit,” tipping the equilibrium from normal development toward malignant transformation.

### 3.2. CXCL10 in OS Proliferation and Motility

The initial growth phase of OS is characterized by elevated CXCL10 expression and activation of CXCL10–CXCR3 signaling within the local TME [17,71]. Multiple cellular sources contribute to this chemokine pool, including OS tumor cells themselves and infiltrating immune populations, particularly tumor-associated macrophages (TAM) [17]. Through autocrine mechanisms, CXCL10–CXCR3 signaling supports OS cell proliferation, survival, and motility, enabling tumor adaptation to the harsh, often hypoxic and nutrient-deprived TME. Experimental evidence underscores the functional significance of this axis. In models employing CRISPR-mediated CXCR3 knockout or pharmacological inhibition, complete loss of CXCR3 markedly suppressed OS proliferation both in vitro and in vivo [71]. Interestingly, pharmacological inhibition produced a milder effect on primary tumor growth but significantly curtailed pulmonary metastasis in both spontaneous and experimental metastasis models [118]. These findings suggest that CXCR3 signaling is essential for OS growth, with particularly pronounced effects during early tumor establishment and metastatic dissemination.

The oncogenic autocrine roles of CXCL10–CXCR3 signaling have been documented across multiple cancer types, including colorectal, breast, prostate, gastric, and melanoma (Table 2) [22]. In colorectal cancer, CXCR3 expression correlates with lymph node metastasis and poor prognosis, where CXCL10 enhances cell survival and invasion partly via matrix metalloproteinase-9 (MMP-9) upregulation [119]. Similarly, CXCL10–CXCR3 signaling promotes lung metastasis in breast cancer [120], lymphatic spread in melanoma [121], and heightened invasiveness in prostate and gastric cancers [95,122], underscoring its conserved pro-metastatic function across malignancies.

Beyond tumor cell–intrinsic signaling, CXCL10 exerts potent paracrine effects within the TME, largely mediated by myeloid populations. In melanoma, CXCL10–CXCR3 crosstalk drives the proliferation and accumulation of immunosuppressive monocytic myeloid-derived suppressor cells (mo-MDSC) via activation of the p38 MAPK pathway, thereby promoting tumor aggressiveness [123]. In hepatocellular carcinoma (HCC), a distinct CXCL10–TLR4–MMP14 signaling axis exacerbates tumor recurrence by mobilizing mo-MDSC. Notably, genetic ablation of either CXCL10 or TLR4 significantly reduces tumor growth and systemic MDSC accumulation [74], illustrating the mechanistic diversity of CXCL10-mediated immunosuppression.

While direct experimental validation of CXCL10’s paracrine roles in OS remains limited, emerging evidence supports a similar immunomodulatory function. Co-culture studies of OS cell lines with THP1-derived M0 or M2 macrophages revealed marked CXCL10 upregulation compared with monocultures, suggesting that CXCL10 contributes to macrophage polarization toward a tumor-promoting M2 phenotype and helps sustain their immunosuppressive activity [17]. The OS TME is notably enriched with naïve macrophages, M2 macrophages, and MDSC—immune subsets that foster tumor progression [17,124]. Given CXCL10’s canonical role as a chemoattractant, local elevation of CXCL10 within OS lesions may also facilitate recruitment of CXCR3^+^ circulating monocytes and macrophages, amplifying the immunosuppressive and tumor-promoting microenvironment [25,125].

**Table 2:** The Dual Roles of the CXCL10-CXCR3 Axis in Cancer Progression and Immune modulation.

Cancer Type	Pro-Metastatic/Pro-Tumor Major Role	Ref.	Anti-Metastatic/Anti-Tumor Major Role	Ref.
Osteosarcoma	CXCR3-A autocrine signaling promotes cell migration, invasion, survival and lung metastasis through the AKT/PAK1 pathway	[71]	High intra-tumoral expression of CXCR3 correlates with dense infiltration of CD8^+^ T cells and NK cells, establishing an immune-hot microenvironment and improved prognosis.	[126]
Breast	Enhances tumor cell motility and promotes metastasis to the lungs and lymph nodes.	[127]	The CXCR3-B isoform has anti-proliferative and anti-angiogenic effects. High axis expression is associated with T-cell infiltration and a better response to therapy.	[69]
Melanoma	Autocrine CXCL10-CXCR3 signaling drives tumor cell proliferation and invasion, contributing to metastasis development and correlating with poor clinical outcomes.	[94]	High intra-tumoral expression of CXCL10 is a key biomarker for a T-cell-inflamed microenvironment and strongly predicts a positive clinical response to anti-PD-1 immunotherapy.	[128]
Colorectal	Promotes cancer cell proliferation, survival, and invasion through activation of the PI3K/AKT and Snail pathways. CXCR3 expression is linked to lymph node metastasis.	[119]	Mediates CD8^+^ T cell facilitation of vessel normalization and improved combinational immunotherapy	[129]
Renal Cell Carcinoma	High CXCR3 expression on tumor cells is associated with advanced tumor grade and metastatic progression.	[70]	In localized disease, high CXCR3 expression is linked to better survival, reflecting a strong, prognostically favorable immune cell infiltrate.	[130]
Pancreatic	Promotes tumor cell migration and contributes to perineural invasion.	[131]	Recruits and maintains an anti-tumor M1 macrophage in the TME. Blockade of the axis accelerates the progression of precancerous lesions.	[25]
Gastric	Promotes invasion and metastasis via the PI3K/AKT pathway	[95]	CXCR3 expression correlates with decreased infiltration of M2 macrophage and favorable outcome	[132]
Hepatocellular Carcinoma (HCC)	Blockade of CXCR3-B signaling increases tumor aggressiveness	[68]	High intra-tumoral expression of CXCL10 and CXCR3 is associated with increased CD8^+^ T-cell infiltration, reduced recurrence, and better overall survival.	[133]
Glioma	Tumoral CXCR3 promotes invasion and progression. Pharmacological antagonism inhibits tumor growth in preclinical models.	[134]	Recruits effector T cells and NK cells across the blood–brain barrier, a critical step for the efficacy of immunotherapies in this cold tumor.	[135]
Prostate	CXCL10-CXCR3 signaling promotes invasion and is associated with bone metastasis.	[122]	High CXCL10 expression within the tumor is associated with increased infiltration of cytotoxic T-cells, decreased Treg, and anti-tumor immunity.	[136]

### 3.3. CXCL10 in OS Pre-Metastatic Niche (PMN) Formation

**Figure 2:**
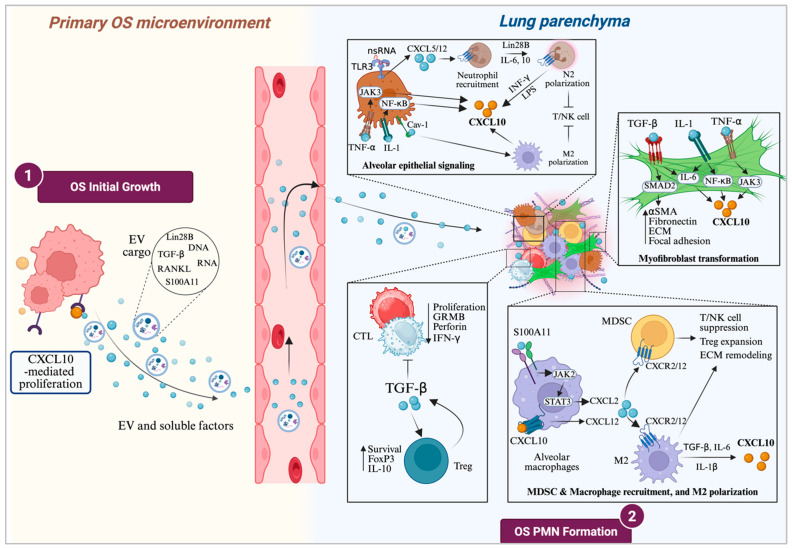
CXCL10 as both a causal mediator and product of lung pre-metastatic niche (PMN) formation. CXCL10–CXCR3 signaling supports initial OS cell proliferation while actively conditioning distant sites by releasing soluble factors and extracellular vesicles (EV) into the circulation. This systemic cargo travels to the lungs, triggering cascades that recruit and reprogram local stromal and immune cells. Tumor-derived TGF-β drives fibroblast-to-myofibroblast transformation and ECM remodeling, while inflammatory cytokines (IL-1, TNF-α) stimulate CXCL10 production. In parallel, alveolar epithelial cells recruit neutrophils and M2 macrophages. Signaling in alveolar macrophages facilitates the recruitment of myeloid-derived suppressor cells (MDSC), establishing an immunosuppressive PMN that suppresses cytotoxic T lymphocyte/NK cell activity and expands Treg populations, creating a permissive microenvironment for metastasis. Standard arrow conventions apply. Created in Biorender. Benjamin B. Gyau. (2025) https://app.biorender.com/illustrations/68cb2e754d9bf3f06d323a5c.

Concurrently with primary tumor growth and prior to metastasis, cancer cells begin to remotely cultivate a supportive microenvironment in distant organs, a process termed pre-metastatic niche (PMN) formation (Figure 2) [137,138]. The formation of the PMN is initiated by signals such as soluble factors and bioactive cargo packaged within extracellular vesicles (EV) released from the primary tumor. While the central concept of PMN formation involves the conditioning of distant organ–resident stromal cells by tumor-derived factors, the specific cellular contributors and molecular mediators vary across tumor types. In colorectal cancer (CRC), for instance, exosomal miR-25-3p acts directly on endothelial cells to increase vascular permeability and angiogenesis, representing a relatively direct mechanism of niche formation [139]. Gastric cancers utilize exosomal miR-519a-3p to polarize intrahepatic macrophages toward a pro-angiogenic M2-like phenotype [140].

In contrast, a more complex, multi-cellular cascade dependent on the CXCL10–CXCR3 axis has been described in melanoma [72] and breast [141] cancer, where it orchestrates a JNK/IL-1–mediated inflammatory and immunosuppressive niche through the intermediary activity of alveolar macrophages or lung-resident fibroblasts. A recurring feature across these diverse models is the critical downstream role of specific immune subsets, particularly monocytic myeloid-derived suppressor cells (mo-MDSC) and M2 macrophages, which are frequently recruited to establish the inflammatory, immunosuppressive, angiogenic, and extracellular matrix (ECM) remodeling conditions necessary for metastatic colonization [142,143].

The precise process of PMN formation in OS remains incompletely understood. However, EV derived from OS cells are known to carry pro-osteoclastogenic factors including TGF-β, MMP-1/13, and RANKL, and can reprogram lung fibroblasts via TGF-β1 signaling toward a pro-metastatic cancer-associated fibroblast (CAF) phenotype [144,145,146]. Additionally, OS-derived, EV-educated MSC promote lung metastasis through TGF-β/IL-6 signaling, and monoclonal antibody blockade of the IL-6 receptor markedly reduces OS pulmonary aggressiveness [147]. Since the TGF-β/IL-6 signaling axis is known to upregulate CXCL10 in inflammatory disease models [148,149,150,151], the CXCL10–CXCR3 axis may represent a central mechanism driving the inflammatory preparation of the lung for the eventual arrival and colonization of circulating OS cells.

We propose that, as OS progresses, the inflammatory milieu established within the developing lung PMN becomes the primary source of the elevated systemic CXCL10 levels observed clinically. This concept is supported by findings from Pein et al. [141], who showed in a breast cancer model that metastatic cells induce lung fibroblasts to upregulate CXCL9 and CXCL10 via JNK/IL-1/NF-κB signaling, fostering an inflammatory microenvironment conducive to tumor colonization. Intriguingly, activation of TGF-β or IL-6 is known to upregulate JNK expression and activity under inflammatory conditions, and vice versa [152,153,154,155], further supporting the downstream activation of the CXCL10–CXCR3 axis as a key pro-inflammatory step orchestrated by OS within the lung. Consequently, this process reframes circulating CXCL10 as a powerful, non-invasive biomarker that not only reflects tumor growth activity but also indicates the active, ongoing reprogramming of the pre-metastatic lung microenvironment and the sustained formation of pulmonary metastases in OS.

## 4. CXCL10’s Anti-Tumor Arm: The Role of CXCL10 in Immune Cell Modulation

### 4.1. CXCL10 in Immune Cell Trafficking

The canonical anti-tumoral function of the CXCL10–CXCR3 axis lies in its capacity to orchestrate adaptive immune responses. Primarily induced by interferon-γ (IFN-γ), elevated CXCL10 levels within the OS TME establish a chemokine gradient that serves as a potent attractant for CXCR3-A-expressing immune effector cells, most notably cytotoxic T lymphocytes (CTL), NK cells, and T helper 1 (Th1) cells [156,157,158]. Ligand binding to CXCR3-A triggers G protein-dependent signaling cascades, including the PI3K and MAPK pathways, which are essential for the cytoskeletal reorganization required for chemotaxis [64,65]. The ensuing infiltration of these anti-tumor immune subsets into the tumor bed constitutes a hallmark of an immunologically hot TME and is frequently associated with a more favorable clinical prognosis [18]. In several malignancies, such as summarized in Table 2 including OS, high intratumoral expression of CXCL10 and/or its receptor CXCR3 correlates with an increased abundance of CD8^+^ T cells, pro-inflammatory M1 macrophages, and activated NK cells, all indicative of a robust anti-tumor immune milieu and improved patient outcomes [25,28,51,126,159]. The ability of CXCL10 to recruit effector immune cells into the TME is therefore a critical determinant of therapeutic efficacy. This is particularly relevant to immune checkpoint inhibitors (ICI), such as anti–PD-1 antibodies, which exhibit maximal benefit in tumors already infiltrated by T cells. For example, elevated CXCL10 levels in melanoma biopsies have been shown to predict a strong clinical response to anti–PD-1 therapy [128]. Similarly, the CXCL10–CXCR3 pathway has been demonstrated to be essential for the synergistic anti-tumor effects observed with combined thermal ablation and PD-1 blockade treatments [133].

### 4.2. CXCL10 in Immune Cell Positioning and Function Modulation

Beyond CXCL10’s role as a chemoattractant for immune cells, it also orchestrates their spatial organization and activation within the TME to ensure an effective anti-tumor response. It has been shown to position recruited T cells in close proximity to CXCL10-producing antigen-presenting cells (APC) such as dendritic cells and M1 macrophages, which is essential for the formation of stable immune synapses and the proper priming and activation of T cells [160]. Accumulating evidence suggests that CXCL10 helps establish a highly structured immune architecture within the tumor; for instance, NK cells and T cells can occupy distinct spatial niches relative to the expression of major histocompatibility complex class I (MHC-I) on tumor cells, indicating that chemokine gradients, including CXCL10, help organize distinct immune compartments [161,162,163]. The efficacy of CXCL10-mediated immune responses may therefore depend not only on the presence of CXCL10 but also on its site of production.

APC-derived CXCL10 throughout the tumor core can promote widespread T-cell infiltration and effective tumor cell killing, whereas production confined to the tumor periphery typically results in a localized, non-infiltrating immune response that fails to control the tumor [163,164,165,166]. This spatial organization represents an immune evasion strategy observed in OS, where immunosuppressive niches enriched with MDSC and M2 macrophages have been identified at the periphery of pulmonary metastases. Intriguingly, the presence of these niches and immune-cold parenchyma correlates with worse progression-free survival [167,168]. While most cancers exhibit an inverse relationship between immunosuppressive cells and anti-tumor immune cells, our recent study of the OS TME and its prognostic significance revealed no such correlation with T cells. Instead, we observed an inverse association between immunosuppressive cells and dendritic cells (DC), which are critical for T cell activation, suggesting that a key immune evasion mechanism in OS may involve interference with CXCL10-mediated recruitment and positioning of DC, effectively dampening T cell-mediated anti-tumor responses [17].

### 4.3. CXCL10 in TME Immunosuppression Modulation

CXCL10’s anti-tumor effects extend beyond attracting adaptive immune cells; it also influences the polarization of TAM. While studies in melanoma and hepatocellular carcinoma suggest that CXCL10 signaling can support a pro-tumor microenvironment dominated by MDSC and macrophages [74,123], findings in early-stage pancreatic cancer indicate a protective role, where CXCL10 was essential for recruiting and maintaining an anti-tumor M1 macrophage population, and its absence caused a shift toward a pro-tumoral M2 phenotype that promotes angiogenesis, tissue remodeling, and metastasis [25]. Previous work from us and others has shown that the OS TME is heavily infiltrated by TAM, which are often skewed toward an immunosuppressive, pro-tumoral M2 phenotype, and we have demonstrated that OS cell lines induce a significant shift to the M2 phenotype when co-cultured with naïve or M1 macrophages [17,30,124]. However, through its interaction with TLR4 on these cells, local CXCL10 can drive their reprogramming toward a pro-inflammatory, anti-tumoral M1 phenotype, thereby shifting the TME from a state that supports tumor growth to one that actively suppresses it [169]. The therapeutic potential of this mechanism has been demonstrated in OS models, where administration of synthetic TLR4 agonists reprogrammed M2 TAM to the M1 phenotype, leading to tumor regression and inhibition of metastasis [77]. Taken together, these findings suggest that CXCL10’s role in modulating innate immunity during OS progression is context-dependent, likely influenced by the specific ligands and chemokine signals present within the TME.

## 5. The Unifying Model: Circulating CXCL10 as a Tumor-Moderated Systemic Immune Decoy in Late-Stage Disease

The contrasting roles of the CXCL10 axis in OS—where its local presence correlates with immune control, yet systemic elevation predicts metastatic burden—necessitate a model that transcends simple binary functions. We propose a unified, dynamic, and spatiotemporal framework that reframes high circulating CXCL10 not merely as a biomarker, but as the functional effector of an OS-derived immune evasion strategy. While elements of this model are supported by direct patient data in OS (e.g., correlation between serum CXCL10 and metastasis and poor survival), the specific kinetic tracking of T-cell misdirection driven by the CXCL10 decoy gradient derive from established paradigms in melanoma and breast cancer biology and remains to be validated in OS models. This model proposes that early tumor priming of the PMN in the lungs, together with local CXCL10-mediated events at the primary tumor, collaboratively establish a systemic CXCL10 gradient that functions as an immune decoy. By doing so, the axis actively subverts the anti-tumor immune response initially established at the primary site, facilitating the transition to overt metastasis.

This process begins concurrently with primary tumor growth during the early stages of metastatic seeding. As immune-evaded OS cells colonize the lungs, they exacerbate the formation of a pro-inflammatory PMN through active reprogramming of key components of the lung microenvironment, including fibroblasts and alveolar macrophages [72,141]. The resulting distant organ inflammation drives a spillover of CXCL10 into the circulation, elevating systemic levels. This transition marks the late stage of the process, wherein the core mechanism of our model—immune misdirection and re-trafficking—becomes dominant. When circulating CXCL10 concentrations surpass those within the primary tumor, a systemic chemokine gradient is established, serving as an immune decoy that intercepts CXCR3-A+-expressing effector cells. This active misdirection prevents cytotoxic T lymphocyte and NK cell accumulation at the primary site, resulting in local immune desertification, a hallmark of aggressive OS that permits unchecked tumor growth [18]. Rather than migrating to the primary tumor to execute their anti-cancer functions, these effector cells are re-routed toward the higher systemic and pulmonary CXCL10 concentration, effectively decoyed away from their intended targets.

Moreover, the receptor profile within the metastatic niche remains a critical variable. While infiltrating T cells and NK cells utilize CXCR3-A for chemotaxis, the metastatic OS cells themselves are selected for high CXCR3-A expression, which drives autocrine survival and colonization signals [60,61,71,118]. This competition for ligand within the metastatic niche, combined with the systemic decoy effect, likely contributes to the immune-desert phenotype often observed in advanced metastatic lesions [170].

This model provides a possible explanation for clinical observations across multiple independent OS patient cohorts. High serum CXCL10 at diagnosis consistently predicts poor overall survival and the development of lung metastases [20,22]. Within this framework, elevated serum CXCL10 is interpreted as a causal consequence of active, ongoing immune misdirection, potentially explaining why OS tumor microenvironments are frequently immune-deserted. Moreover, it reconciles why high intra-tumoral CXCR3 expression correlates with favorable prognosis: in the absence of a dominant systemic decoy, high local CXCR3 signifies a well-infiltrated, immune-hot OS capable of mounting an effective anti-tumor response [126]. Conversely, in the presence of a systemic CXCL10 gradient, OS tumors that produce chemoattractants fail to recruit adequate immunity, compromising this potent immune-recruiting system and tipping the balance toward unchecked tumor growth and overt metastasis.

## 6. Therapeutic Implications and Future Directions

### 6.1. Targeting the Pro-Metastatic Arm: CXCR3 Inhibition

**Figure 3:**
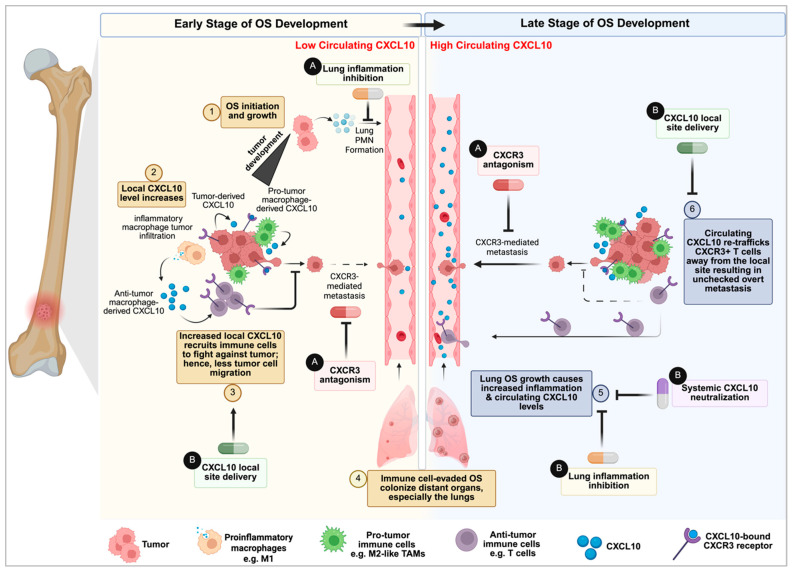
Working model of the CXCL10-CXCR3 axis in OS. Locally, tumor-derived CXCL10 promotes OS growth via autocrine CXCR3 signaling while recruiting CXCR3-A^+^ immune cells for anti-tumor responses. However, lung colonization induces inflammation that amplifies systemic CXCL10, creating a gradient that acts as an immune decoy, misdirecting T and NK cells away from the primary tumor. Two therapeutic strategies are proposed: (A) CXCR3 signaling inhibition using antagonists or degraders to disrupt the pro-tumor feedback loop and suppress metastasis; and (B) A combined approach enhancing local CXCL10 availability (e.g., oncolytic viruses, nanoparticles) while neutralizing circulating CXCL10 levels and lung inflammation, thereby restoring effective anti-tumor immune cell infiltration. See main text for details for the working model and therapeutic strategies. Standard arrow conventions apply. Created in Biorender. Benjamin B. Gyau. (2025) https://app.biorender.com/illustrations/66bd2d5c18fa5796ae15dba7.

Our proposed model and a better understanding of the role of CXCL10 in OS pathogenesis opens several promising avenues for therapeutic development. For patients identified early (e.g., with low to medium circulating CXCL10 and no overt metastases), a logical strategy is to disrupt the entire pro-tumor cycle. Systemic administration of a CXCR3 antagonist, such as AMG487, could confer dual benefits by inhibiting the direct pro-migratory effects on OS cells and simultaneously preventing CXCL10-mediated conditioning of lung macrophages, thereby blocking PMN formation and the subsequent establishment of the systemic immune decoy. Preclinical evidence supporting this approach is robust (Figure 3-A), suggesting that early intervention targeting the CXCL10–CXCR3 axis may effectively mitigate both primary tumor progression and the facilitation of metastatic dissemination [71,118,120,171].

#### 6.1.1. CXCR3 Antagonism

The use of small molecule CXCR3 antagonists has demonstrated significant efficacy in OS models, markedly reducing tumor cell migration in vitro and substantially inhibiting the development of lung metastases in vivo [71,118]. The utility of this approach is not limited to OS: AMG487, for example, has shown promise in preclinical models of breast cancer, where it decreases metastasis while simultaneously enhancing host anti-tumor immunity, and in glioma, where it prolongs survival [120,134]. While a key concern is the potential for creating an immune-depleted TME by blocking beneficial anti-tumor immune cell trafficking, evidence suggests this may not be prohibitive [118,120,172]. Given that most OS tumors are considered immune-cold [173], CXCR3 antagonists may produce a strong anti-metastatic effect with minimal negative impact on an already limited immune response.

Despite broad preclinical success, the translation of CXCR3 antagonists into clinical oncology has faced substantial challenges. Development of AMG487 and other antagonists, including SCH 546738, NBI-74330, and TAK-779, failed to demonstrate efficacy in clinical trials, largely due to unfavorable drug properties rather than flaws in the biological hypothesis [174]. For instance, a retrospective analysis of early clinical trials with agents like AMG487 in metastatic renal carcinoma and other solid tumors suggests that these failures may stem from the non-selective inhibition of both CXCR3-A and CXCR3-B isoforms [174,175]. Pan-inhibition may inadvertently abrogate the angiostatic and apoptotic signals mediated by CXCR3-B, neutralizing the therapeutic benefit [71,176]. Consequently, the future of this therapeutic class in OS depends on the development of isoform-selective antagonists or allosteric modulators that specifically disrupt CXCR3-A signaling while sparing CXCR3-B [177]. Next-generation antagonists such as ACT-777991, with improved pharmacodynamics and pharmacokinetics, warrant renewed investigation [178,179,180].

#### 6.1.2. CXCR3 Degradation

In our recent report studying the effects of different inhibitory strategies targeting the CXCR3 axis on OS aggressiveness, we observed a mechanistically important distinction between the effects of genetic deletion versus pharmacological inhibition of CXCR3. While both approaches effectively impaired metastasis, the genetic absence of the receptor from the outset produced a markedly stronger inhibitory effect on primary tumor proliferation and growth than acute pharmacological blockade [71]. While this could reflect a distinct role for CXCR3 during early tumor development, our data point towards a more fundamental mechanistic difference between the two inhibitory approaches. The observed significant effect of the KO suggests that ligand-independent signaling functions of the CXCR3 protein, which are unaffected by pharmacological inhibition, play a critical role in OS proliferation in vitro. Therefore, this finding suggests that therapies designed to degrade the CXCR3 protein, such as proteolysis-targeting chimeras (PROTAC), may be more effective in achieving a comprehensive anti-tumor effect compared to conventional antagonists [181,182]. Conversely, in patients with established primary tumors where the immediate clinical challenge is to prevent metastatic dissemination, CXCR3 antagonists that block ligand-dependent signaling remain a logical and validated therapeutic strategy.

#### 6.1.3. Dual CXCR3/CXCR4 Antagonism

The CXCL10–CXCR3 axis does not operate in isolation but is part of a complex network of chemokine signaling pathways that collectively regulate cell fate and migration. Of particular importance is its crosstalk with the CXCL12–CXCR4 axis, another master regulator of tumor progression and metastasis in OS and numerous other cancers, which is known to promote OS proliferation and lung metastasis [183,184]. Emerging evidence indicates significant interplay between these two axes: CXCR3 and CXCR4 can form functional heterodimers on the cell surface, potentially altering their signaling properties [185,186], and in colorectal cancer cells, CXCR3 expression has been shown to prevent CXCR4 internalization, thereby sustaining and enhancing its pro-invasive signaling output [187]. These observations suggest a cooperative or synergistic relationship in which the two axes act in concert to drive an aggressive metastatic phenotype. This has critical therapeutic implications, as targeting only one receptor may lead to compensatory upregulation or continued signaling through the other, whereas a strategy involving simultaneous blockade of both CXCR3 and CXCR4 could provide a more robust and durable anti-metastatic effect.

### 6.2. Harnessing the Anti-Tumor Arm: Immunotherapy and Recruitment

In contrast to strategies aimed at inhibiting CXCL10’s pro-tumor effects, a second major therapeutic paradigm is to harness its potent immune-recruiting capabilities. This approach is particularly relevant for immunologically cold tumors like OS, which are often characterized by a lack of T-cell infiltration and are consequently resistant to many forms of immunotherapy.

#### 6.2.1. Primary Site CXCL10 Upregulation

By increasing the local concentration and activity of CXCL10 within the TME (Figure 3-B), it may be possible to convert this immune desert into a hotbed of anti-tumor activity. Promising strategies include oncolytic viruses engineered to express CXCL10, which have shown efficacy in reprogramming the TME in other cancers by providing a sustained, high-concentration local chemokine source [188]. Bispecific T-cell engagers (TCE) and NK-cell engagers (NKCE) act as molecular bridges linking immune effector cells (T cells or NK cells) to tumor cells to trigger targeted killing; while highly effective in hematological malignancies, their success in solid tumors is often limited by insufficient effector cell trafficking and infiltration into the dense, immunosuppressive TME [189,190,191]. Since both T cells and NK cells express CXCR3, increasing local CXCL10 can serve as a homing signal, recruiting these effector cells to sites where engager molecules can mediate cytotoxicity. For example, in glioblastoma, a multifunctional NKCE that binds the activating receptor NKp46 on NK cells, a tumor-specific antigen on GBM cells, and incorporates a domain that locally releases CXCL10, significantly enhanced CXCR3^+^ NK cell recruitment and anti-tumor activity [192], illustrating its potential application in OS.

As the field progresses, advanced trispecific TCE combining tumor targeting, T-cell engagement (anti-CD3), and co-stimulation (anti-CD28) or checkpoint inhibition (anti-PD-1) could be further potentiated by co-administering therapies that upregulate local CXCL10, transforming recruitment from a passive to an active process and overcoming a major barrier in solid tumor immunotherapy [163,188].

#### 6.2.2. Circulating CXCL10 Neutralization

Because high circulating CXCL10 functions as a systemic decoy that redirects anti-tumor immune cells away from the primary site, a complementary therapeutic approach is the systemic neutralization of this chemokine using monoclonal antibodies (mAb) (Figure 3-B). Preclinical evidence strongly supports this approach in the context of bone malignancies. In a mouse model of bone metastasis, systemic treatment with a neutralizing anti-CXCL10 antibody significantly reduced the migration of CXCR3-expressing cancer cells to the bone and decreased overall tumor burden [110]. This suggests that neutralizing the ligand can effectively disrupt the metastatic cascade. Furthermore, clinical-grade fully human anti-CXCL10 monoclonal antibodies, such as MDX-1100 (eldelumab), have already been developed and tested in Phase II clinical trials for rheumatoid arthritis and ulcerative colitis [193]. These trials demonstrated that systemic CXCL10 neutralization is well-tolerated in humans, providing a tangible pathway for repurposing these agents for metastatic OS.

This strategy may be particularly effective when employed in a combinatorial setting. By pairing systemic neutralization with therapies that specifically induce local CXCL10 production (such as oncolytic viruses or localized radiotherapy as described above), clinicians could artificially generate a steep chemotactic gradient that strongly favors immune infiltration to the primary tumor site. This dual strategy addresses a critical barrier in treating cold tumors like OS and could potentially be extrapolated to other malignancies where high serum CXCL10 correlates with poor prognosis, such as melanoma and hepatocellular carcinoma [188,194,195,196,197].

### 6.3. Maximizing the Efficacy of CXCR3 and CXCL10 Modulators

To maximize the therapeutic potential of modulating CXCL10–CXCR3 signaling, strategies that combine multiple treatment modalities and leverage advanced delivery platforms for enhanced precision and efficacy are warranted. The complex biology of the CXCL10 axis, with its opposing functions, makes it a good candidate for combination therapies and sophisticated delivery systems that can exert spatial and temporal control over chemokine signaling, immune recruitment, and tumor targeting.

#### 6.3.1. Combination Therapy

The success of immune checkpoint blockade (ICB) therapies, such as antibodies targeting PD-1, PD-L1, and CTLA-4, is critically dependent on the presence of a pre-existing T-cell infiltrate within the tumor, as tumors lacking this infiltrate are typically resistant to ICB [198,199]. The CXCL10–CXCR3 axis has emerged as a central mechanistic driver of ICB efficacy: unbiased analyses of the TME following dual PD-1/CTLA-4 blockade revealed CXCL10 as one of the most significantly upregulated chemokines, with its signaling through CXCR3 essential for therapeutic effect [162]. Single-cell RNA sequencing of patient tumors has identified M1 TAM as the predominant source of CXCL10 both before and after ICB therapy, and the presence of a distinct macrophage signature expressing these chemokines is strongly associated with positive clinical responses [162]. For OS, strategies that induce local CXCL10 production are critical to sensitize tumors to ICB, potentially through combination with therapies that induce inflammatory responses, such as radiation, thermal ablation, or agents that activate innate immune pathways (e.g., TLR or STING agonists). Additionally, measuring baseline intratumoral CXCL10 or the presence of CXCL10-producing TAM could serve as predictive biomarkers to identify OS patients most likely to benefit from ICB. An especially promising approach is a triple combination with CXCR3-targeted therapies, which could reduce OS metastatic capacity while enhancing susceptibility to locally boosted CXCL10-mediated immune attack.

#### 6.3.2. Targeted Delivery

Nanoparticle (NP) technology provides a versatile platform to overcome challenges associated with targeting the CXCL10 axis, including poor bioavailability, off-target toxicity, and achieving high therapeutic concentrations within the TME [200,201]. NP can be employed for localized delivery of pathway inhibitors; for example, in a murine model of inflammatory bone loss relevant to OS, local injection of the CXCR3 antagonist AMG487 encapsulated in nanoparticles effectively reduced inflammation, decreased osteoclast numbers, and prevented bone resorption, demonstrating the potential to disrupt the pro-tumor vicious cycle without systemic toxicity [202]. Similarly, nanoparticle-based CXCL10 stimulators are being developed to enhance ICB efficacy in melanoma, further highlighting their clinical applications [203]. Conversely, NP can deliver immunostimulatory cargo: a nanocapsule platform successfully packaged and delivered the T-cell-attracting chemokine CCL21, enhancing immune infiltration and tumor growth inhibition in lung cancer models [204], a strategy readily adaptable for CXCL10 to convert immune-cold tumors into hot ones. Furthermore, since CXCL10 can modulate the physical tumor barrier, treatment with TNFα can increase vascular permeability, facilitating the extravasation of NP-encapsulated chemotherapy into the tumor [205], underscoring the versatility of this approach in combination with chemokine-based therapies.

### 6.4. Validation and Biomarkers

#### 6.4.1. Validating the Spatiotemporal Model

An immediate priority is validating this spatiotemporal model in preclinical systems capable of tracking immune cell flux between the primary tumor, circulation, and the lung PMN in real time. Specifically, the use of photoconvertible immune cell reporters (e.g., Kaede or KikGR mice) and longitudinal in vivo imaging could be employed to definitively demonstrate the sequestration of CXCR3+ effector cells in the lungs or circulation in response to tumor-driven CXCL10 gradients [206,207]. Moreover, emerging technologies such as spatial transcriptomics and multiplexed ion beam imaging (MIBI) may offer unprecedented opportunities to map CXCL10 gradients relative to the locations of immune cells within the OS TME and lung PMN, providing important visual and quantitative evidence [208,209,210]. A disconnect between high CXCL10 expression and low T-cell density in the primary tumor, contrasted with high T-cell accumulation in CXCL10-rich pulmonary niches, would provide strong correlative evidence for the decoy mechanism.

#### 6.4.2. Novel Biomarker Strategies: Beyond Serum CXCL10

While high serum CXCL10 is an established negative prognostic marker [20,211], the complexity of the axis suggests that ligand levels alone may not capture the full biological picture. Future clinical studies should investigate the expression profile of CXCR3 isoforms (CXCR3-A vs. CXCR3-B) on circulating immune cells. A high frequency of CXCR3-A+ effector cells in the blood, in the presence of elevated serum CXCL10, may confirm the immune decoy status, indicating that while the host has generated effector cells, they are being misdirected. Conversely, a downregulation of CXCR3-A on peripheral T cells could indicate T-cell exhaustion or desensitization. Developing standardized flow cytometric assays to quantify these isoform ratios in patient PBMCs could provide a more granular prognostic tool than serum chemokine levels alone.

## 7. Conclusions

The clinical trajectory of OS has remained frustratingly stagnant for decades, largely due to its aggressive metastasis to the lungs. This review has sought to address a central paradox in OS biology: the dual and conflicting roles of the chemokine CXCL10. Moving beyond the simplistic “good versus bad” narrative, we propose a unified, dynamic, and spatiotemporal model that reconciles these observations. Our model reframes CXCL10 not merely as a biomarker but as a functional, systemic modulator of immune evasion, a process initiated by the primary OS tumor and its early communication with the PMN in the lungs.

We have systematically deconstructed how the CXCL10 axis operates on multiple fronts. Locally, within the primary tumor, CXCL10 can exert potent anti-tumor effects by recruiting cytotoxic T cells and NK cells, consistent with its favorable prognostic value when confined to the TME. However, this local benefit is subverted by systemic processes. The concurrent formation of an inflammatory PMN in the lungs creates a dominant, alternative source of CXCL10, leading to a gradual and then overwhelming rise in circulating CXCL10 levels. This high systemic concentration establishes a chemokine gradient that functions as an immune decoy, actively misdirecting CXCR3^+^ tumor-specific immune cells away from the primary tumor. The resulting immune desert allows the primary tumor to thrive unchecked while continuing to shed metastatic cells, providing a mechanistic explanation for why high circulating CXCL10 is a robust predictor of poor survival.

This refined understanding, integrating OS cell-intrinsic signaling, PMN conditioning, and systemic immune trafficking, offers a roadmap for future research and therapeutic design. Immediate priorities include validating this spatiotemporal model in preclinical systems capable of tracking immune cell flux between the primary tumor, circulation, and the lung PMN in real time. Key unanswered questions remain, including the molecular triggers that initiate CXCL10 production in the PMN and the contribution of alternative CXCL10 receptors, such as ACKR2, in shaping chemokine gradients. Addressing these questions will deepen our understanding of how systemic chemokine signaling orchestrates immune evasion in metastatic OS. Furthermore, future clinical studies should evaluate whether the ratio of CXCR3-A to CXCR3-B expression on circulating immune cells and tumor cells, combined with serum CXCL10 levels, serves as a more precise prognostic indicator of metastatic susceptibility.

Most importantly, this model provides a strategic framework for designing therapies tailored to the patient’s CXCL10 profile and local immune context. For patients with high circulating CXCL10, indicative of compromised local immunity, therapeutic strategies should focus on disrupting the systemic chemokine signal, potentially through combinations of CXCR3 antagonists and agents targeting the inflammatory PMN. Conversely, in patients with immune-cold tumors and low systemic CXCL10, the goal should be to re-establish local immune dominance, achievable with approaches such as the use of CXCL10-expressing oncolytic viruses and nanoparticle-loaded CXCL10 to generate a localized chemokine gradient that recruits a robust anti-tumor response. By targeting the cycle of inflammation, systemic misdirection, and immune evasion orchestrated by CXCL10, this framework has the potential to transform the treatment paradigm for metastatic OS, converting a master multi-facet regulator into a viable therapeutic target.

## Figures and Tables

**Table 1:** Functional Dichotomy of CXCR3 Major Isoforms in OS.

Feature	CXCR3-A	CXCR3-B
Primary Function	Pro-tumorigenic, Pro-metastatic, Chemotaxis	Anti-tumorigenic, Angiostatic, Pro-apoptotic
Signaling Pathways	Gαi-dependent; PI3K/AKT, MAPK/ERK, Ca^2+^ mobilization	Gαs-dependent; cAMP/PKA, p38 MAPK
Key Cellular Targets	Tumor Cells: Promotes survival, proliferation, invasion. T/NK Cells: Drives chemotaxis/recruitment	Tumor Cells: Induces growth arrest/apoptosis. Endothelial Cells: Inhibits migration/tube formation.
Role in OS	Overexpressed in metastatic OS; drives lung colonization via PAK1 activation.	Often downregulated in metastatic OS; restoration inhibits growth.
Ligand Affinity	High affinity for CXCL9, CXCL10, CXCL11	High affinity for CXCL4 (PF-4), CXCL9, CXCL10, CXCL11
Therapeutic Implications	Target for antagonism (inhibit metastasis)	Target for agonism (restore angiostasis/apoptosis)

## Data Availability

No new data were created or analyzed in this study. Data sharing is not applicable to this article.

## Abbreviations

The following abbreviations are used in this manuscript:

OS	Osteosarcoma
CXCL10	C-X-C Motif Chemokine Ligand 10
CXCR3	C-X-C Motif Chemokine Receptor 3
TLR4	Toll-Like Receptor 4
ACKR2	Atypical Chemokine Receptor 2
IFN-γ	Interferon-γ
TME	Tumor Microenvironment
ELR	Glutamic Acid-Leucine-Arginine
PMN	Pre-Metastatic Niche
MSC	Mesenchymal Stem Cells
TAM	Tumor-Associated Macrophages
MDSC	Myeloid-Derived Suppressor Cells
CAF	Cancer-Associated Fibroblasts
APC	Antigen-Presenting Cells
DC	Dendritic Cells
CTL	Cytotoxic T Lymphocytes
EMT	Epithelial–Mesenchymal Transition
EV	Extracellular Vesicles
ICB	Immune Checkpoint Blockade
ICI	Immune Checkpoint Inhibitors
NP	Nanoparticles
PAK1	p21-Activated Kinase 1
PROTAC	Proteolysis-Targeting Chimeras
RANKL	Receptor Activator of Nuclear factor-κB Ligand
TCE	T Cell Engagers
NKCE	Natural Killer-Cell Engagers
